# The Golden Age of Pauperism

**Published:** 1886-12-04

**Authors:** 


### THE GOLDEN AGE OF PAUPERISM.

A Man of the Times.

The Rev. S. A. Barnett, Vicar of St. Jude's, Whitechapel, is a well-known "man of the times." Perhaps most clear-headed and honest-hearted men who pursue their daily work among the people of the East-end of London are compelled to be "men of the times." Among the rich and prosperous a minimum of sense and business capacity may be combined with a maximum of what Carlyle has called "solemn plausibility," without exciting any very serious comment, or without anybody finding out that there is a sounding brass or a tinkling cymbal in their midst. But Mr. Barnett, as well as many another, has found out that at the East-end everybody must "mean business." If we were Irish we should say, "So much the better; more power to him."

Bumbledom and the Paupers.

The Vicar of St. Jude's has proposed, but the Whitechapel Guardians have disposed, and disposed contrary to the Vicar's proposal. The business which the Guardians have been called upon to consider is not peculiar to Whitechapel, but concerns every board of guardians in the kingdom, and not only so but every medical man, every pauper, and every other person. Mr. Barnett says three things: first, that the art of medicine has made very rapid progress within the last two or three decades; secondly, that the paupers have not had their share of the advantages of that progress; and thirdly, that they ought to have had, or, at any rate, that they ought now to be put in the way of getting their share of these advantages. We are inclined to think that the answer to these proposals would be an excellent test of a man's whereabouts as a Christian and as a statesman. Bumbledom will no doubt say that paupers ought to be thankful to get any medical treatment at all, and not to complain because they do not get the very best. As for the "highest kind of nursing," which Mr. Barnett also insists upon, that they certainly have no right to expect any more than the other. Now all this sounds quite natural and reasonable, but Mr. Barnett is not a man of very limited experience. He has been long and favourably known to the public as a most admirable parish clergyman, and any sober proposal which he considers it desirable to make is worthy of the fullest consideration.

"Do the Right."

Mr. Barnett proposes that in addition to the *resident* medical officers at the Whitechapel Union, there shall be one or more *visiting* physicians and surgeons, and that these shall be men of undoubted scientific acquirements, and proved practical ability. Thus, says he, the interior of the workhouse infirmary would be in touch with the active and progressive spirit of the best medical science of the age. On what ground does he base his revolutionary proposal? On the noblest of all grounds—because it is *right.* If you give paupers medical treatment at all, he says in effect, then give them the best. Such a proposal could only have come from a man who recognises in every other human being a brother man or a sister woman. A different type of man would have said: "If you do this you will convert your poor-law infirmary into a large medical school, and by the multiplication of such schools you will give vastly increased facilities to medical men for the gaining of a broader and deeper knowledge, and so you will benefit the whole community by improving the quality of its medical men." And that would have been a very convincing argument to use, more convincing, perhaps, to the average guardian's mind than that actually employed by Mr. Barnett. Indeed, we ourselves, looking at the question from a public point of view, attach great importance to that argument. Still, we hold with the vicar of St. Jude's, that the first and most convincing reason in favour of his proposal is that it is right, and therefore it should be done.

Ways and Means.

Of course, in the application of such a proposal there are many difficulties. You must first catch your scientific visiting physicians and surgeons; and how are you going to catch them? There are plenty of them, no doubt, but are you going to do as the general hospitals do, and to obtain the very highest professional skill the country affords without fee or reward? There is no mention made of ways and means, but it seems to us that a proposal to increase the number of high-class men doing purely gratuitous work is one which requires more than a passing consideration. Mr. Barnett's idea itself is good, and the movement altogether in the right direction, but if it becomes successful we hope it will not be at the cost of expensively educated and hard-working but often very impecunious doctors. Probably a certain number of men may be found who would take such appointments as are proposed without payment. But, we ask, is it fair that a rich country like ours should tempt men to serve it in this way, and offer them nothing but some shadowy possibility of future success in practice as their reward? Still, whatever be the difficulties, the proposal itself meets with our emphatic approval. If the paupers are to have medical attendance let them have the best; and if the Poor-law Infirmaries be, as they undoubtedly are, admirable fields for the higher study of medical science and practice, let them be thrown open to all those who desire to prosecute their studies until the highest perfection is reached. The paupers would no doubt be the immediate gainers by the change, but within a very short time the medical profession and the whole community would be repaid a hundredfold for any little additional cost which the carrying out of the proposal might entail. Mr. Barnett has been defeated this time, but we hope he will return to the attack and prosecute it with intelligence and renewed vigour to a successful issue.

